# Correlation between expression of p53, p21/WAF1, and MDM2 proteins and their prognostic significance in primary hepatocellular carcinoma

**DOI:** 10.1186/1479-5876-7-110

**Published:** 2009-12-22

**Authors:** Mei-Fang Zhang, Zhi-Yi Zhang, Jia Fu, Yu-Feng Yang, Jing-Ping Yun

**Affiliations:** 1 State Key Laboratory of Oncology in Southern China, Cancer Center of Sun Yat-Sen University, Guangzhou, China; 2 Department of Pathology, Cancer Center, Sun Yat-Sen University, Guangzhou 510060, China

## Abstract

**Background:**

Tumor Protein p53 (p53), cyclin-dependent kinase inhibitor 1A (p21/WAF1), and murine double minute 2 (MDM2) participate in the regulation of cell growth. Altered expression of these gene products has been found in malignant tumors and has been associated with poor prognosis. Our aim was to investigate the expression of the 3 proteins in hepatocellular carcinoma (HCC) and their prognostic significance.

**Methods:**

We examined p53, p21/WAF1, and MDM2 expression in 181 pairs of HCC tissues and the adjacent hepatic tissues by performing immunohistochemistry and examined the expression of the 3 proteins in 7 pairs of HCC tissues and the adjacent hepatic tissues by using western blot analysis.

**Results:**

The expression of p53, p21/WAF1, and MDM2 in the HCC tissues was significantly higher than those in the adjacent hepatic tissues (*P *< 0.05). A statistical correlation was observed between p53 and p21/WAF1 expression in HCC tissues (R = 0.195, *P *= 0.008). A statistical correlation was observed between expression of p53 and p21/WAF1 (R = 0.380, *P *= 0.000), p53 and MDM2 (R = 0.299, *P *= 0.000), p21/WAF1 and MDM2 (R = 0.285, *P *= 0.000) in 181 liver tissues adjacent to the tumor. Patients with a low pathologic grade HCC (I+II) had a higher tendency to express p53 on tumor cells than the patients with high pathologic grade HCC (III+IV) (*P *= 0.007). Survival analysis showed that positive p21/WAF1 expression or/and negative MDM2 expression in HCC was a predictor of better survival of patients after tumor resection (*P *< 0.05).

**Conclusions:**

The proteins p53, p21/WAF1, and MDM2 were overexpressed in all the HCC cases in this study, and p53 and p21/WAF1 overexpression were positively correlated. The expression of p21/WAF1 and MDM2 can be considered as 2 useful indicators for predicting the prognosis of HCC.

## Background

Hepatocellular carcinoma (HCC) is the fifth most common malignancy worldwide and is the third most common cause of cancer-related deaths [1]. HCC develops in patients with chronic liver diseases, and its etiopathogenesis includes viral infection (hepatitis B and C), alcohol, and aflatoxin B1 consumption. The majority of HCC patients have associated cirrhosis and impaired liver function, making the treatment of HCC more difficult than that of many other cancers. Surgery, including transplantation, remains the only potential curative modality for HCC.

Prognosis of HCC remains unsatisfactory even after surgical resection and liver transplantation. Considerable interest has been generated in identifying factors that influence the prognosis of HCC. Several staging systems have been developed to predict survival period after the diagnosis of HCC [2]. The most widely studied prognostic factors are related to the pathological characteristics of the neoplasm, including tumor size, grade, stage, and vascular invasion. However, several biological molecules that can predict the survival period of HCC patients have been reported in recent years; however, the results are controversial.

Previous studies have explored the molecular alterations in HCC, including changes in the expression of p53, cyclin-dependent kinase inhibitor 1A (p21/WAF1), and murine double minute 2 (MDM2). The tumor suppressor gene *p53 *plays a key role in regulating the cell cycle and serves as a principal mediator of growth arrest, senescence, and apoptosis in response to a broad array of cellular damage [3]. The p21/WAF1 protein is encoded by the human *WAF1/CIP1 *gene and its expression is directly induced by the wild-type p53 protein [4]. This protein binds to a variety of cyclin-dependent kinases and inhibits their activity, regulates DNA repair, and directly blocks DNA replication by inhibiting the proliferating cell nuclear antigen [5], thus inhibiting cell-cycle progression and decreasing cell growth. MDM2 is the product of a p53-inducible gene and inhibits the p53 activity by ubiquitinating p53 and creating a negative-feedback loop [5-8]. Altered expression of these gene products has been found in malignant tumors including HCC and correlated with poor prognosis. In HCC, the prognostic value of p53 is controversial, since several studies show an association of p53 with patient survival [9-12], while other investigations report no association [13,14]. The predictive value of the p21/WAF1 expression level in HCC is also ambiguous [10,11,15]. However, few studies pertaining to the expression of the 3 proteins p53, p21/WAF1, and MDM2 in HCC cases have reported different results [11,16].

We determined the expression of p53, p21/WAF1, and MDM2 in a relatively large sample size of 181 pairs of human HCC tissues and the corresponding adjacent hepatic tissues obtained after resection by performing immunohistochemistry (IHC). In addition, we performed western blot analysis in 7 such pairs. Further, we attempted to address the correlation among their expression and the relationship between their expression and the clinical parameters, including overall survival.

## Methods

### Clinical samples

Samples from 181 Chinese patients with HCC and their clinical records from 1997 to 2007 were collected from the Cancer Center of Sun Yat-Sen University, Guangzhou, China. Tissue blocks prepared from HCC tissues and the adjacent liver tissues were sectioned for performing IHC of p53, p21/WAF1, and MDM2; in addition, for 7 cases, we collected the tissue samples inclusive of the HCC and its adjacent tissues from the tissue bank department of this cancer center and subjected these samples to western blot analyses. The collection of the human specimens in the study was approved by the Independent Ethics Committee of the Cancer Center of Sun Yat-Sen University.

### Western blot analysis

For immunolabeling, lysates were prepared from the tissues as described previously [17,18]. We separated 100 μg of each lysate by sodium dodecyl sulfate-polyacrylamide gel electrophoresis (SDS-PAGE). The proteins were transferred onto blotting membranes. After blocking, the membranes were incubated overnight with rabbit polyclonal antibody against p53 (Clone: FL-393; Cat No. sc-6243; Santa Cruz, CA); mouse monoclonal antibody against p21/WAF1 (Clone: SX118; Cat No. 556430; BD Pharmigen, CA) and MDM2 (Clone: N-20; Cat No. sc-813; Santa Cruz, CA); and mouse monoclonal antibody against glyceraldehydes 3-phosphate dehydrogenase (GAPDH) (Kangchen Biotech; Shanghai, China) (p53, 1:500; p21/WAF1, 1:250; MDM2, 1:2000; and GAPDH, 1:1000), followed by incubation with horseradish peroxidase-conjugated immunoglobulin G (IgG). The blots were then visualized by using an ECL kit (Amersham Life Science; Piscataway, NH, USA) and exposed for 1 min to an X-ray film.

### Immunohistochemistry

For immunohistochemistry studies, a labeled-streptavidin-biotin (LAB-SA) method was performed with Histostain^®^-*P*lus Bulk Kit Zymed^® ^2^nd ^generation LAB-SA detection system (CAT. NO. 85-9043, Zymed Laboratories, CA) as previously described [18,19]. All the primary antibodies (p53, p21/WAF1, and MDM2; mouse monoclonal antibody, Cat No. ZM-0408, ZM-0206, and ZM-0425, respectively, Zymed, CA) were ready to use without dilution. Each paraffin-embedded tissue section (4 μm in thickness) was deparaffinized, hydrated, and incubated in 3% H_2_O_2 _and microwaved for 3 minutes to block endogenous peroxidase activity. The tissue sections were subjected to antigen retrieval by microwaving in 10 mM citrate buffer for 30 min. The sections were incubated with serum blocking solution (Reagent A) for 10 minutes to block nonspecific binding and then with the primary antibodies in moist chamber for 60 minutes. After rinsed with PBS for 2 minutes, the sections were incubated with the biotinylated secondary antibody (Reagent B) for 10 minutes and rinsed with PBS. The sections followed by incubation with enzyme conjugate (Reagent C) for 10 minutes. Subsequently, the sections were stained with DAB and counterstained with hematoxylin. Serum blocking solution (Reagent A) in place of the primary antibody was used as a negative control. A brown particle in nuclei was considered as positive labeling. Immunostaining labeling intensities were defined as: +, less than 10% of the tumor cells were positive; ++, 10%-50% of the tumor cells were positive; +++, more than 50% the tumor cells were positive; -, negative staining. These sections were observed under light microscopy and the staining intensities were assessed by 2 pathologists--Dr JP Yun and Dr MF Zhang.

### Statistical analysis

Statistical analysis was performed to determine the relationship between the clinical parameters of gender, age, tumor size, number of tumors, hepatitis B surface antigen (HBsAg), pathologic grade, serum level of alpha-fetal protein (AFP), and the 3 immunohistochemical markers by Peason's chi-square test. The Spearman correlation was employed to examine the relationship between the expression of p53, p21/WAF1, and MDM2. Survival was assessed by the Kaplan-Meier method, and log-rank test was used to analyze survival curves. Statistical significance was initially set at *P *< 0.05. All statistical analysis was performed using the SPSS 13.0 software for Windows.

## Results

### Increase in the expression of p53, p21/WAF1, and MDM2 in HCC

**Figure 1:**
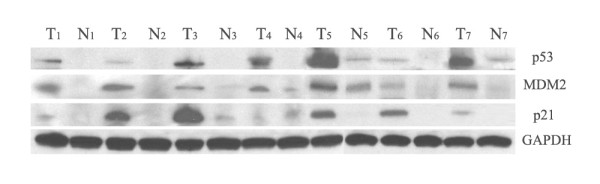
**Expression of p53, p21/WAF1 and MDM2 in HCC by Western blot**. The expression of p53, p21/WAF1, and MDM2 was detected in hepatocellular carcinoma (HCC) tissues by western blot analysis. We used 7 pairs of HCC tissues and the adjacent hepatic tissues. Tissues T1-7 were HCC tissues and N1-7 were the adjacent hepatic tissues. The expression of the housekeeping gene, glyceraldehydes 3-phosphate dehydrogenase (GAPDH), served as a control. The expression of p53 was higher in the HCC tissues (T1-7) than in the adjacent hepatic tissues (N1-7). The MDM2 expression followed a similar trend in both the tissues. The expression of p21/WAF1 was higher in HCC tissues (T1-3, T5-7) than the adjacent hepatic tissues (N1-3, N5-7).

The expression of p53 and MDM2 in the 7 pairs was higher in the HCC tissues than in the adjacent hepatic tissues (tissues 1-7), as determined by western blot (Figure 1). In 6 out of 7 pairs, p21/WAF1 expression was higher in the HCC tissues than in the adjacent hepatic tissues (tissues 1-3 and 5-7). In 1 case, the expression of p21/WAF1 in the HCC tissue was lower than that in adjacent hepatic tissue (tissue 4). These results indicated that the expression levels of p53, p21/WAF1, and MDM2 were higher in the HCC tissues than those in the adjacent hepatic tissues.

**Figure 2:**
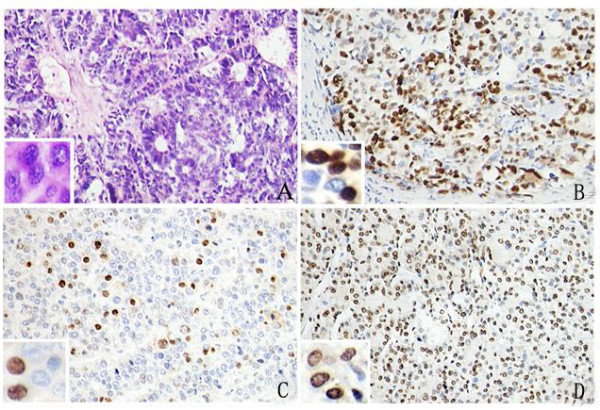
**Expression of p53, p21/WAF1 and MDM2 in HCC by IHC**. The hematoxylin and eosin (H&E) stained sections show a solid area of hepatocellular carcinoma (HCC) (A). Immunohistochemical staining for p53 (B), p21/WAF1 (C), and MDM2 (D) in HCC. (Mag. ×400).

The expression of p53, p21/WAF1, and MDM2 in the 181 pairs of tissues was analyzed by IHC. As shown in Figure 2, p53, p21/WAF1, and MDM2 were mainly located in the nuclei of the cancer cells and highly expressed in the HCC tissue. Statistical analysis showed that positive proportions of p53, p21/WAF1, and MDM2 expression in HCC tissues were 70.7% (128/181), 33.1% (60/181), and 52.5% (95/181), respectively. Positive proportions of p53, p21/WAF1, and MDM2 expression in the corresponding adjacent hepatic tissues were 16.6% (30/175), 15.5% (28/178), and 32.6% (59/179), respectively. The expression of p53, p21/WAF1, and MDM2 in HCC was significantly higher than that in adjacent hepatic tissues (*P *< 0.05 for each protein).

### Statistically significant correlation between p53, p21/WAF1, and MDM2 expression in HCC tissues

We calculated the correlation between p53, p21/WAF1, and MDM2 expression in 181 HCC tissues by Spearman correlation analysis (Table 1). Statistical correlation was observed between p53 and p21/WAF1 expression in HCC (R = 0.195, *P *= 0.008). No statistical correlations were observed between p53 and MDM2 expression in HCC (*P *= 0.058) and between p21/WAF1 and MDM2 expression in HCC (*P *= 0.431). Interestingly, statistical correlations were observed between the expressions of p53 and p21/WAF1 (R = 0.380, *P *= 0.000), p53 and MDM2 (R = 0.299, *P *= 0.000), p21/WAF1 and MDM2 (R = 0.285, *P *= 0.000) in 181 liver tissues adjacent to the tumor (Table 2).

**Table 1:** Correlation among p53, p21/WAF1, and MDM2 expression in HCC tissues

	n	p53 positive		p21/WAF1 positive		MDM2 positive	
		
		n	*P *value	n	*P *value	n	*P *value
p53	181						
positive	128			50	0.008*	73	0.058
negative	53			10		22	
p21/WAF1	181						
positive	60	50	0.008*			34	0.431
negative	121	78				61	
MDM2	181						
positive	95	73	0.058	24	0.431		
negative	86	55		18			

*Statistically significant (*P *< 0.05, 2-sided probability)

**Table 2:** Correlation among p53, p21/WAF1, and MDM2 expression in the adjacent hepatic tissues

	n	p53 positive		p21/WAF1 positive		MDM2 positive	
		
		n	*P *value	n	*P *value	n	*P *value
P53	175						
positive	30			14	0.000*	19	0.000
negative	145			14		40	
p21/WAF1	178						
positive	28	13	0.000*			18	0.000
negative	150	17				41	
MDM2	179						
positive	59	18	0.000	18	0.000		
negative	120	12		10			

*Statistically significant (*P *< 0.05, 2-sided probability)

We further investigated the differences between the expression of p53, p21/WAF1, and MDM2 in 181 pairs of HCC on the basis of different clinical parameters, including the gender, age, tumor size, number of tumors, HBsAg, pathologic grade, and serum level of AFP of the patient. We observed a statistical correlation between p53 and the pathologic grade in HCC tissues (*P *= 0.007). Patients with a low pathologic grade (I+II) had a higher tendency to express p53 on tumor cells than patients with high pathologic grade (III+IV). No statistical significance was found between p53, p21/WAF1, and MDM2 expression and the other clinical parameters (Table 3).

**Table 3:** The expression of p53, p21/WAF1, and MDM2 in HCC tissues and clinical parameters

	Cases(n)	p53 positive		p21/WAF1 positive		MDM2 positive	
		n (%)	*P *value	n (%)	*P *value	n (%)	*P *value
Sex	181						
Male	165	117(70.9)	0.857	53(32.1)	0.348	84(50.9)	0.174
Female	16	11(68.8)		7(43.8)		11(68.8)	
Age	181						
<45 y	75	55(73.3)	0.518	23(30.7)	0.553	38(50.7)	0.682
≥45 y	106	73(68.9)		37(34.9)		57(53.8)	
Tumor size	181						
<5 cm	61	45(73.8)	0.523	21(34.4)	0.796	33(54.1)	0.758
≥5 cm	120	83(69.2)		39(32.5)		62(51.7)	
Tumor amount	181						
1	145	100(69.0)	0.301	46(31.7)	0.417	74(51.0)	0.435
≥2	36	28(77.8)		14(38.9)		21(58.3)	
HbsAg	181						
Positive	161	116(72.0)	0.267	53(32.9)	0.853	85(52.8)	0.815
Negative	20	12(60.0)		7(35.0)		10(50.0)	
Histological grade^Δ^	181						
I+II	143	108(75.5)	0.007*	49(34.3)	0.627	75(52.4)	0.940
III+IV	38	20(52.6)		11(28.9)		206(52.6)	
Serum AFP	181						
<20 ng/ml	52	37(71.2)	0.935	22(42.3)	0.098	26(50.0)	0.673
≥20 ng/ml	129	91(70.5)		38(29.5)		69(53.5)	

^Δ^Histological grade was with reference to the World Health Organization classification published in 2002.

*Statistically significant (*P *< 0.05, 2-sided probability)

### Positive p21/WAF1 expression or/and negative MDM2 expression in HCC tissues associated with better survival in patients

**Figure 3:**
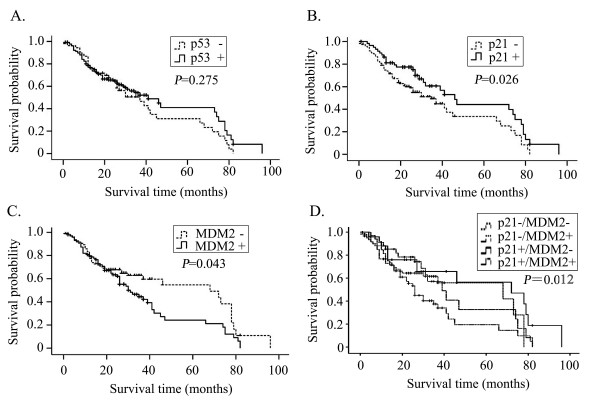
**The Kaplan-Meier survival curves**. The Kaplan-Meier survival of p53, p21/WAF1 and MDM2 in HCC. Positive or negative p53 expression did not correlate with the survival of patients (*P *= 0.275) (A). There was a significant difference in survival between patients with positive p21/WAF1 expression and negative p21/WAF1 expression (*P *= 0.026) (B) and between patients with negative MDM2 expression and positive MDM2 expression (*P *= 0.043) (C). There was a significant difference in survival between patients with p21+/MDM2- expression and p21-/MDM2+ expression (*P *= 0.012) (D).

The associations between survival time and the 3 immunohistochemical markers (p53, p21/WAF1, and MDM2) in HCC were analyzed with Kaplan-Meier survival analysis (Figure 3). The survival curve for p21/WAF1-positive patients tended to be better than that for p21/WAF1-negative patients (*P *= 0.026). The survival curve for MDM2-negative patients tended to be better than that for MDM2- positive patients (*P *= 0.043). There was no significant correlation between p53 expression and the survival time of the patients (*P *= 0.275). Further analysis of the prognostic value of combining p21 and MDM2 expression in HCC was undertaken. It can be divided into 4 groups: p21+/MDM2-, p21+/MDM2+, p21-/MDM2- and p21-/MDM2+. The survival curve for p21+/MDM2- patients tended to be better than that for p21-/MDM2+ patients (*P *= 0.012), and there was no significant difference between the other groups. These results indicated that the expression of p21/WAF1 and MDM2 were associated with survival in patients with HCC.

## Discussion

The results from our study revealed a significant increase in the expression of p53, p21/WAF1, and MDM2 in HCC tissues than the corresponding adjacent hepatic tissues; the expression levels of the 3 proteins in the former was 70.7%, 33.1%, and 52.5%, respectively and those in the later were 16.6%, 15.5%, and 32.6%, respectively. These results indicated that these proteins play important roles in hepatocarcinogenesis.

Several IHC-based studies have reported the proportion of p53-positive HCC cases to vary in the range of 22% to 81% [20]. The cause for the variation in p53 expression can be partly attributed to the lack of a consistent cutoff value among different studies for determining positive p53 expression. In some studies, the HCC was regarded as p53-positive when ≥10% of the tumor cells expressed p53, while in others, this cutoff value was defined as ≥5% of the tumor cells being positive for p53; further, the majority of studies have not defined the lower limit for p53-positive tumor cells. Another cause of the discrepancy in the reported percentage of p53-positive tumors is the differences in the p53 expression with the prevalent carcinogenic factors and certain unknown molecular mechanisms. The tumor suppressor gene, *p53*, has been reported to be mutated in 24-69% of HCCs. Mutations of *p53 *result in unregulated replication of defective DNA, genomic instability, and cancer progression because of the loss of the tumor-suppressive activity of the wild-type *p53 *gene. Wild-type *p53 *has a short half-life and is therefore undetectable by IHC. Mutations in the *p53 *gene result in stabilization of the protein, permitting nuclear accumulation, and immunohistochemical detection. A number of previous studies have focused on the incidence of *p53 *gene mutations or p53 protein expression in HCC and have reported that there is a large variation among geographical areas because of the differences in the prevalent carcinogenic factors and some unknown molecular mechanisms. However, few of studies have investigated the p53 protein expression in the liver tissues adjacent to the tumor in the same group of HCC patients. On the basis of our results, the comparison between p53 expression in HCC tissues and the corresponding adjacent liver tissues indicate that IHC can be used to assess the status of p53 expression in HCC and that p53 plays important roles in hepatocarcinogenesis.

The protein p21/WAF1 plays a key role in the p53-mediated cell cycle arrest in response to DNA damage [5,21-23]. Its expression varies in different malignancies; it is overexpressed in non-small cell lung carcinoma [24] and cutaneous squamous cell carcinoma [25], but is decreased in colorectal carcinoma [26] and ovarian carcinoma [27]. Previous studies have suggested that p21/WAF1 mRNA expression in nontumor liver tissues is significantly higher than that in HCC tissues, indicating that its expression might represent a form of cyclin dependent kinase (CDK) inhibitor dysfunction involved in tumorigenesis. However, Qin [28] reported much higher expression of p21/WAF1 in HCC tissues (64.9%) than in the corresponding adjacent liver tissues (30.9%) by IHC. In another report, more than 90% cases showed negative staining for p21/WAF1 in nontumor liver tissues [15]. Similar to Qin's observation, we observed a significant difference of p21/WAF1 expression between the HCC tissues and the corresponding adjacent liver tissues. Overexpression of p21/WAF1 in HCC tissues cannot be attributed to its mutation since no mutant forms of p21/WAF1 have been detected thus far. A possible explanation for the overexpression of p21/WAF1 is that aberrant CDK-inhibitory regulation leads to incomplete inhibition of CDK activity and suppresses tumor cell growth, which probably results in increased expression of the protein so as to control the abnormal cell-cycle progression and suppress the replication of tumor cells [28]. Overexpression of p21/WAF1 can be considered to be a late-stage molecular event in hepatocarcinogenesis.

Contrary to some previous reports [15,28], our data showed that p53 expression correlated with p21/WAF1 expression either in HCC tissues or in corresponding adjacent liver tissues, indicating that both p53 and p21/WAF1 may play a role in hepatocarcinogenesis. Correlation between the expression of p21/WAF1 and p53 in nontumor liver tissues is expected because p21/WAF1 activation in nontumor hepatic areas occurs in a p53-dependent manner [29-31]. However, the correlation between their expression in HCC tissues is unexpected. We hypothesize that the expression of the protein p21/WAF1 in HCC tissues may also be activated in a p53-dependent manner; however, additional experiments should be performed to confirm this hypothesis. MDM2 and p53 are linked to each other through an autoregulatory negative feedback loop aimed at maintaining low cellular p53 levels in the absence of stress [8]. Mutant p53 proteins in tumor cells are stable because they are deficient in transactivating MDM2 -- hence they have a defective negative feedback loop [8]. These can explain our results that p53 expression correlates with MDM2 expression in corresponding adjacent liver tissues but not in HCC tissues.

The results from our study showed that the expression of p21/WAF1 and MDM2 in HCC was associated with survival in patients with HCC, indicating that p21/WAF1 and MDM2 can be considered as predictors of the prognosis of HCC patients. Previous studies have reported conflicting results on the prognostic value of p53, p21/WAF1, and MDM2 expression in HCC. Naka [9] reported that positive immunostaining for p53 protein expression was a significant indicator of poor prognosis in 126 HCC patients. Further, Sung [12] reported that p53 overexpression was a poor prognostic factor of survival in 105 HCC patients, and Schoniger-Hekele [11] reported that the survival of patients overexpressing p53 among 81 HCC patients was poorer than that of those who did not express p53. Hu [32] reported that patients overexpressing p53 among 124 HCC patients had shorter survival periods and higher recurrence rates than the p53-negative patients. In a study conducted using samples collected from 122 HCC patients after tumor resection, Kao [15] reported that HCC patients with negative expression of p21/WAF1 exhibited a poorer survival rate than the HCC patients with positive expression of p21/WAF1, suggesting that the expression of p21/WAF1 in HCC was a survival prognostic factor. MDM2 was rarely reported to be a prognostic factor in HCC, but was often reported in the maxillary sinus squamous cell carcinoma [33], in Egyptian esophageal carcinoma [34], in breast carcinoma [35], prostate adenocarcinoma [36], gastric cancer [37,38], and epithelial ovarian cancer [39,40].

## Conclusion

In summary, we provided evidence for the significant higher expression of p53, p21/WAF1, and MDM2 in HCC tissues than in the corresponding adjacent liver tissues; p53 expression correlated with p21/WAF1 expression either in HCC tissues or in corresponding adjacent liver tissues. Further, we determined that p21/WAF1 and MDM2 expression in HCC was associated with survival in patients with HCC, indicating that p21/WAF1 and MDM2 can be used to predict the prognosis of patients with HCC.

## Competing interests

The authors declare that they have no competing interests.

## Authors' contributions

JPY carried out and coordinated the study, immunohistochemical examinations of tumor specimens and data analysis, and drafted the manuscript. ZYZ and JF participated in the interpretation of data, conducted immunohistochemistry, and western blot analysis. All authors read and approved the final manuscript.
